# Use of nouns and verbs in the oral narrative of individuals with hearing impairment and normal hearing between 5 and 11 years of age

**DOI:** 10.1590/1516-3180.2013.1315384

**Published:** 2013-10-01

**Authors:** Érica Endo Amemiya, Bárbara Niegia Garcia Goulart, Brasilia Maria Chiari

**Affiliations:** I Speech-Language Pathologist, Communication Disorders, Hospital São Paulo, Universidade Federal de São Paulo (Unifesp), São Paulo, Brazil.; II MSc, PhD. Adjunct Professor, Social Psychology Department, Universidade Federal do Rio Grande do Sul (UFRGS), Porto Alegre, Brazil.; III PhD. Titular Professor, Department of Speech- Language and Hearing Sciences, Universidade Federal de São Paulo (Unifesp), São Paulo, Brazil.

**Keywords:** Child language, Language development, Deafness, Communication disorders, Narration, Linguagem infantil, Desenvolvimento da linguagem, Surdez, Transtornos da comunicação, Narração

## Abstract

**CONTEXT AND OBJECTIVE:**

Nouns and verbs indicate actions in oral communication. However, hearing impairment can compromise the acquisition of oral language to such an extent that appropriate use of these can be challenging. The objective of this study was to compare the use of nouns and verbs in the oral narrative of hearing-impaired and hearing children.

**DESIGN AND SETTING:**

Analytical cross-sectional study at the Department of Speech-Language and Hearing Sciences, Universidade Federal de São Paulo.

**METHODS:**

Twenty-one children with moderate to profound bilateral neurosensory hearing impairment and twenty-one with normal hearing (controls) were matched according to sex, school year and school type. A board showing pictures was presented to each child, to elicit a narrative and measure their performance in producing nouns and verbs.

**RESULTS:**

Twenty-two (52.4%) of the subjects were males. The mean age was 8 years (standard deviation, SD = 1.5). Comparing averages between the groups of boys and girls, we did not find any significant difference in their use of nouns, but among verbs, there was a significant difference regarding use of the imperative (P = 0.041): more frequent among boys (mean = 2.91). There was no significant difference in the use of nouns and verbs between deaf children and hearers, in relation to school type. Regarding use of the indicative, there was a nearly significant trend (P = 0.058).

**CONCLUSION:**

Among oralized hearing-impaired children who underwent speech therapy, their performance regarding verbs and noun use was similar to that of their hearing counterparts.

## Abstract (pt)

**CONTEXTO E OBJETIVO:**

Substantivos e verbos indicam ações na comunicação oral. Na ocorrência de perda auditiva, a aquisição da linguagem oral pode ser comprometida a tal ponto que o uso apropriado deles pode constituir importante desafio. O objetivo deste estudo é comparar o uso de substantivos e verbos na narrativa oral de deficientes auditivos e de ouvintes.

**TIPO DE ESTUDO E LOCAL:**

Estudo transversal analítico no Departamento de Fonoaudiologia, Universidade Federal de São Paulo.

**MÉTODOS:**

21 crianças portadoras de deficiência auditiva neurossensorial de grau moderado a profundo bilateral e 21 crianças com audição normal (controles) pareadas por sexo, ano de escolaridade e tipo de escola. Uma prancha com figuras foi apresentada para cada criança para elicitar uma narrativa e medir o desempenho na produção de substantivos e verbos.

**RESULTADOS:**

22 (52,4%) eram do sexo masculino. A idade média foi de 8 anos (desvio padrão, DP = 1,5). Ao comparar a média entre os grupos de meninos e meninas, não encontramos diferença significativa no uso de substantivos, porém entre os verbos, encontramos diferença significante no uso do imperativo (P = 0,041): mais frequente entre os meninos (média = 2,91). Não houve diferença significativa no uso de substantivos e verbos entre crianças surdas e ouvintes, em relação ao tipo de escola. No caso do uso do modo indicativo, houve uma tendência a diferença (P = 0,058).

**CONCLUSÕES:**

Crianças com deficiência auditiva oralizadas e submetidas à fonoterapia obtiveram desempenho semelhante a de seus pares ouvintes quanto ao uso de verbos e substantivos.

## INTRODUCTION

Language acquisition is a complex process based on neurological and psychosociocultural maturation and is an important element in the human learning process._^1^_ Correct use of language also requires the ability to integrate verbal and semantic components through intonation and expression in communication._^2^_ Furthermore, language is the form of communication that allows expression through speech and consists of a code to be learned._^1^_

Speech and language are fundamental means for social communication. In speaking or writing, there is a social purpose. Language mediated by code turns humans into relational beings. For this reason, hearing impairment is an important challenge in relation to language acquisition, given the importance of oral language acquisition._^3-5^_ Although monitoring may occur, its efficiency is not always satisfactory._^4^_

### OBJECTIVES

The aim of the present study was to show the importance of nouns and verbs in language acquisition among deaf children. Nouns have the purpose of showing the world and verbs provide action in events, and both of these are important categories in oral discourse. Thus, the present study compared the use of nouns and verbs in the oral narrative of hearing-impaired and hearing children between the ages of 5 and 11 years.

### METHODS

This study was appraised and approved by the Research Ethics Committee of Hospital São Paulo. Forty-two children of both genders between the ages of 5 and 11 years took part in this casecontrol study: 21 were neurosensory hearing-impaired children (Experimental group: impaired hearing) who had acquired moderate to profound degrees of bilateral hearing impairment before reaching the age of three years and starting speech-language therapy. They were using personal sound amplification devices and predominantly used oral linguistic code to communicate (none of them were sign language users). The control group (hearing subjects) included 21 children whose hearing was within the normal parameters, without complaints concerning communication or learning, who were matched according to age, sex and educational level with the hearing-impaired children. The sample in this study presented sufficient statistical power (95%): 18 patients were needed for this study in each group, as calculated using Compare_^2^_ (WinPepi software) with a sample size ratio of 1:1 and differences between means ranging from -0.54 to +0.54, and without any sample loss._^6^_

To elicit the oral narrative, the sequence of pictures called "The Dog's Story" by Le Boeuf_^7^_ was shown by the evaluator to each child, individually, on a single board with the pictures placed in the correct sequence. Each child was asked to tell the story orally, from the sequence and also to give it a title.

The children were given as much time as they needed to become acquainted with the pictures and to start the narrative. Whenever they were ready, the evaluator recorded the narratives individually using a Sony digital camera (model Cybershot DSCW30). During the narrative, the children were allowed to have visual and tactile contact with the picture board. The images were then recorded on a computer and a canonical transcription of each child's speech was made.

To analyze the transcribed narratives, the frequencies of nouns (people, things, places and ideas, in terms of, collective, simple, compound, derivate and primitive nouns) and of verbs (indicativo, subjuntivo, imperativo, presente, pretérito perfeito, pretérito imperfeito, pretérito mais que perfeito, futuro do pretérito and futuro do presente) were considered.

Comparisons between the experimental and control groups were made according to the mean, standard deviation and median. Moreover, the variables of the child's age, severity of hearing loss, age at which prosthesis use started and educational level were analyzed in relation to their narrative performance, in both the experimental group (impaired hearing) and the control group (hearing subjects). For the statistical analysis, the independent t-test was used to compare the means of the variables and their associations (Student's t test) between the hearing-impaired group and the hearers, with the significance level set at P < 0.05 (5%).

### RESULTS

Among the children evaluated, 22 (52.4%) were males. The mean age was 8 years (standard deviation, SD = 1.5). Concerning the educational level, as measured by the number of years of schooling, the mean was 4.8 years (standard deviation, SD = 1.4). The mother's educational level was, on average, 10.9 years (standard deviation, SD = 4.3). Among the 42 children evaluated, 22 (52.4%) were attending private schools. Hearing loss among the 21 deaf children was, on average, detected at the age of 38.8 months (standard deviation, SD = 26.9; median = 36 months). The mean age at which they started to use a prosthesis was 53.5 months (SD = 30.3; median = 60 months) and the mean time that had elapsed between the beginning of speech-language therapy and the present evaluation was 41.9 months (SD = 34.4; median = 48 months). The average hearing loss for the best ear was 71.2 decibels (SD = 18).

The use of nouns and verbs was assessed based on the participants' gender. Table 1 shows the results from independent t-tests comparing the average noun usage of the groups of boys and girls. No significant differences were found in relation to any of the nouns analyzed, based on the participants' gender. Table 2 shows the results from the independent t-test comparing the average verb usage of the groups of boys and girls. A significant difference was found regarding the use of the imperative (Student's t test, t = 2.117; degrees of freedom, gl = 40; P = 0.041).

**Table 1:** Descriptive data and independent t test for comparison of noun type use in relation to the participant's sex

Noun type	Sex	n	Mean	Standard deviation	Standard error	t	df	Sign
SP	MaleFemale	2319	1.090.79	2.112.51	0.440.58	0.418	40	0.678
SC	Male Female	23 19	18.87 17.89	6.93 6.31	1.45 1.45	0.472	40	0.639
SCO	Male Female	23 19	18.3017.53	6.65 6.10	1.39 1.40	0.392	40	0.697
SA	Male Female	23 19	0.91 0.95	0.79 1.13	0.17 0.26	-0.115	40	0.909
SCOL	Male Female	23 19	0.000.00	0.00 0.00	0.00 0.00	0*	0*	0*
SS	Male Female	23 19	18.5717.95	6.65 6.70	1.39 1.54	0.299	40	0.767
SCOM	Male Female	23 19	0.830.53	0.78 0.61	0.16 0.14	1.366	40	0.180
SD	Male Female	23 19	1.521.84	1.73 2.57	0.36 0.59	-0.481	40	0.633
SPR	Male Female	23 19	17.8716.68	6.70 5.47	1.40 1.25	0.619	40	0.539

SP = proper noun

SC = common noun

SCO = concrete noun

SA = abstract noun

SCOL = collective noun

SS = simple noun

SCOM = compound noun

SD = derivative noun

SPR = primitive noun

* = significant difference

t = Student's t test

df = degrees of freedom

Sign = significance

**Table 2:** Descriptive data and independent t test for comparison of verb type use in relation to the participant's sex

Verb tense	Sex	n	Mean	Standard deviation	Standard error	t	df	Sign
Infinitivo	Male Female	23 19	1.96 1.53	1.82 1.12	0.38 0.26	0.897	40	0.375
Gerúndio	Male Female	23 19	0.13 0.05	0.34 0.23	0.07 0.05	0.842	40	0.405
Particípio	Male Female	23 19	12.43 12.11	4.12 3.68	0.86 0.84	0.271	40	0.788
Indicativo	Male Female	23 19	0.26 0.05	0.54 0.23	0.11 0.05	1.564	40	0.126
Subjuntivo	Male Female	23 19	0.57 0.63	1.04 0.83	0.22 0.19	-0.225	40	0.823
Imperativo	Male Female	23 19	2.91 1.11	3.42 1.59	0.71 0.37	2.117	40	0.041*
Presente	Male Female	23 19	7.48 8.95	4.08 3.14	0.85 0.72	-1.287	40	0.206
Pretérito Perfeito	Male Female	23 19	2.26 2.05	1.96 2.27	0.41 0.52	0.319	40	0.751
Pretérito Imperfeito	Male Female	23 19	0.00 0.00	0.00 0.00	0.00 0.00	0*	0*	0*
Pretérito mais que perfeito	Male Female	23 19	0.00 0.00	0.00 0.00	0.00 0.00	0*	0*	0*
Futuro do pretérito	Male Female	23 19	0.00 0.00	0.00 0.00	0.00 0.00	0*	0*	0*
Futuro do presente	Male Female	23 19	0.13 0.05	0.46 0.23	0.10 0.05	0.673	40	0.505

* = significant difference

t = Student's t test

df = degrees of freedom

Sign = significance

According to the data, boys (mean = 2.91 times) used the imperative more frequently than the girls did (mean = 1.11 times). The use of nouns and verbs was also assessed based on type of school (public versus private). Table 3 shows the comparison between the types of school attended by the participants in relation to their use of nouns, which did not find any significant difference. A similar comparison was performed (Table 4) for the use of verbs, which also showed no significant difference, although we observed a nearly significant trend (Student's t test, t = 1.948; degrees of freedom, gl = 40; P = 0.058) for simple indicative tense use. The children who were attending public schools tended to use the simple tense more often (mean = 0.30) than the students at private schools did (mean = 0.05). Tables 5 and 6 show comparisons between the hearing-impaired and control groups regarding their use of nouns and verbs, which did not find any significant differences. The interactions between independent variables for groups of verbs and nouns were also tested, using multivariate GLM (General Linear Model), but no significant differences were found (Wilks' lambda = 0.525; F17.18 = 0.957; significance, P = 0.534; power, Po = 0.393).

**Table 3:** Descriptive data and independent t test for comparison of noun type use in relation to the type of school that the participants attended

Noun type	School type	n	Mean	Standard deviation	Standard error	t	df	Sign
SP	Public Private	20 22	1.25 0.68	2.88 1.55	0.64 0.33	0.805	40	0.425
SC	Public Private	20 22	16.70 20.00	5.97 6.88	1.33 1.47	-1.653	40	0.106
SCO	Public Private	20 22	16.60 19.18	6.13 6.43	1.37 1.37	-1.330	40	0.191
SA	Public Private	20 22	0.95 0.91	1.10 0.81	0.25 0.17	0.138	40	0.891
SCOL	Public Private	20 22	0.00 0.00	0.00 0.00	0.00 0.00	0*	0*	0*
SS	Public Private	20 22	16.95 19.50	6.45 6.64	1.44 1.42	-1.260	40	0.215
SCOM	Public Private	20 22	0.65 0.73	0.59 0.83	0.13 0.18	-0.346	40	0.731
SD	Public Private	20 22	1.30 2.00	2.27 1.98	0.51 0.42	-1.067	40	0.292
SPR	Public Private	20 22	16.30 18.27	5.55 6.60	1.24 1.41	-1.043	40	0.303

SC = common noun

SCO = concrete noun

SA = abstract noun

SCOL = collective noun

SS = simple noun

SCOM = compound noun

SD = derivative noun

SPR = primitive noun

* = significant difference

t = Student's t test

df = degrees of freedom

Sign = significance

**Table 4:** Descriptive data and independent t test for comparison of verb type use in relation to the type of school that the participants attended

Verb tense	School type	n	Mean	Standard deviation	Standard error	t	df	Sign
Infinitivo	Public Private	20 22	1.70 1.82	1.38 1.71	0.31 0.36	-0.245	40	0.808
Gerúndio	Public Private	20 22	0.10 0.09	0.31 0.29	0.07 0.06	0.098	40	0.923
Particípio	Public Private	20 22	11.85 12.68	3.91 0.30	0.87 0.83	-0.689	40	0.495
Indicativo	Public Private	20 22	0.30 0.05	0.57 0.21	0.13 0.05	1.948	40	0.058
Subjuntivo	Public Private	20 22	0.75 0.45	1.02 0.86	0.23 0.18	1.019	40	0.314
Imperativo	Public Private	20 22	1.95 2.23	2.95 2.86	0.66 0.61	-0.309	40	0.759
Presente	Public Private	20 22	7.85 8.41	4.06 3.45	0.91 0.73	-0.483	40	0.632
Pretérito Perfeito	Public Private	20 22	22.30 2.05	2.20 2.01	0.49 0.43	0.391	40	0.698
Pretérito Imperfeito	Public Private	20 22	0.00 0.00	0.00 0.00	0.00 0.00	0*	0*	0*
Pretérito mais que perfeito	Public Private	20 22	0.00 0.00	0.00 0.00	0.00 0.00	0*	0*	0*
Futuro do pretérito	Public Private	20 22	0.00 0.00	0.00 0.00	0.00 0.00	0*	0*	0*
Futuro do presente	Public Private	20 22	0.05 0.14	0.22 0.47	0.05 0.10	-0.751	40	0.457

* = significant difference

t = Student's t test

df = degrees of freedom

Sign = significance

**Table 5:** Descriptive data and independent t test for comparison of noun type use in relation to the hearing-impaired (HI) and control groups

Noun type	Group	n	Mean	Standard deviation	Standard error	t	df	Sign
SP	HI Control	21 21	0.43 1.48	1.25 2.91	0.27 0.63	-1.517	40	0.137
SC	HI Control	21 21	19.71 17.14	8.03 4.61	1.75 1.01	1.273	40	0.211
SCO	HI Control	21 21	18.76 17.14	7.40 5.13	1.62 1.12	0.824	40	0.415
SA	HI Control	21 21	0.95 0.90	1.02 0.89	0.22 0.19	0.161	40	0.873
SCOL	HI Control	21 21	0.00 0.00	0.00 0.00	0.00 0.00	0*	0*	0*
SS	HI Control	21 21	18.95 17.62	7.92 5.06	1.73 1.11	0.650	40	0.519
SCOM	HI Control	21 21	0.76 0.62	0.70 0.74	0.15 0.16	0.643	40	0.524
SD	HI Control	21 21	1.62 1.71	2.18 2.12	0.48 0.46	-0.143	40	0.887
SPR	HI Control	21 21	18.19 16.48	6.94 5.22	1.51 1.14	0.905	40	0.371

SC = common noun

SCO = concrete noun

SA = abstract noun

SCOL = collective noun

SS = simple noun

SCOM = compound noun

SD = derivative noun

SPR = primitive noun

HI = impaired hearing

* = significant difference

t = Student's t test

df = degrees of freedom

Sign = significance

**Table 6:** Descriptive data and independent t test for comparison of verb type use in relation to the hearing-impaired (HI) and control groups

Verb tense	Group	n	Mean	Standard deviation	Standard error	t	df	Sign
Infinitivo	HI Control	21 21	2.00 1.52	1.79 1.25	0.39 0.27	1.000	40	0.323
Gerúndio	HI Control	21 21	0.10 0.10	0.30 0.30	0.07 0.07	0.000	40	1.000
Particípio	HI Control	21 21	12.57 12.00	4.87 2.65	1.06 0.58	0.472	40	0.639
Indicativo	HI Control	21 21	0.10 0.24	0.30 0.54	0.07 0.12	-1.061	40	0.295
Subjuntivo	HI Control	21 21	0.62 0.57	0.97 0.93	0.21 0.20	0.162	40	0.872
Imperativo	HI Control	21 21	2.29 1.90	2.33 3.37	0.51 0.74	0.426	40	0.672
Presente	HI Control	21 21	7.90 8.38	3.99 3.50	0.87 0.76	-0.411	40	0.683
Pretérito Perfeito	HI Control	21 21	2.29 2.05	2.55 1.53	0.56 0.33	0.367	40	0.716
Pretérito Imperfeito	HI Control	21 21	0.00 0.00	0.00 0.00	0.00 0.00	0.830	40	0.411
Pretérito mais que perfeito	HI Control	21 21	0.00 0.00	0.00 0.00	0.00 0.00	0*	0*	0*
Futuro do pretérito	HI Control	21 21	0.00 0.00	0.00 0.00	0.00 0.00	0*	0*	0*
Futuro do presente	HI Control	21 21	0.14 0.05	0.48 0.22	0.10 0.05	0.830	40	0.411

* = significant difference

t = Student's t test

df = degrees of freedom

Sign = significance

### DISCUSSION

Many variables influence the acquisition and development of speech in the presence of hearing deficits, such as congenital or acquired loss, loss level type, time of diagnosis, time of sensory deprivation, etiology, use of electronic devices, school and family. Hearing loss makes the acquisition and development of speech more difficult in terms of form, content and use._^5^_ Thus, it is important that individuals with impaired hearing should always keep their memory system active in order to maintain the ability to perceive and retain the, memory of ideas, their organization and their structure._^8^_

In analyzing the grammatical development of children and the growth of their vocabulary, it is necessary to consider that nouns and verbs have different characteristics._^9^_ Nouns usually correspond to the name given to something or someone and frequently work as an argument, whereas verbs usually express actions and processes and have the characteristic of working as a predicate._^10^_

Verbs have wide semantic and grammatical variety, which prevents easy generalization. Children need to be exposed several times to the same verb, for them to learn its properties. Because verb references are not as clear as noun references, verb acquisition usually occurs in a gradual manner._^10^_

Several parameters were studied to characterize the sampled individuals' production. First, we approached the findings based on the gender parameter: the sample consisted of 22 males and 19 females. The results from the independent t-test comparing the averages between the groups of boys and girls showed no significant differences for any of the nouns analyzed, as shown in Table 1. In relation to the use of verbs, a significant difference in the use of the imperative was found (P = 0.041). According to the average values, the boys (mean = 2.91) used the imperative more frequently than the girls did (mean = 1.11). In the specialized literature, we did not find any studies on the influence of gender on the acquisition of nouns and verbs, with regard to comparing hearing-impaired and hearing subjects._^11-14^_ Therefore, the idea of the importance of appropriate biological apparatus and an adequate environment for the development of speech is strengthened._^15^_

The analysis on the proportion of nouns showed no significant difference between the two types of schools analyzed. Table 3 does not show any significant difference between the use of nouns according to the school type and the same was seen in relation to the use of verbs according to school type, as shown in Table 4. However, in the same table, a nearly significant trend (P = 0.058) was observed in relation to the use of the simple tense indicative. Students in public schools (P = 0.30) were shown to use the simple tense more often than did students in private schools (P = 0.05).

The relationship between socioeconomic status (measured according to the type of school attended) and speech acquisition among hearing-impaired children depends on external circumstances (stress and limitations) and internal factors (attitude and conventional style). These elements influence the diversity of a child's speech._^16^_ Importantly, the quality and frequency of stimuli that the child receives from the environment may not be associated with the family's socioeconomic level._^17^_ Social interaction, among other factors that do not depend exclusively on financial status, is an enriching tool for acquisition of narrative structure, which benefits children in relation to organizing time, space, causal relations, cohesion and coherence. With training in these abilities, construction of writing will be effective._^17^_

Tables 5 and 6 show that there were no significant differences in the presence of hearing impairment, relating either to nouns or to verbs. The interactions between the independent variables for groups of verbs and nouns were tested using multivariate GLM, but no statistically significant differences were found (P = 0.534).

The simple present tense and the simple past were the most frequently used tenses in the oral narratives of both the hearing and the hearing-impaired children. These results confirm that the simple past and perfect past predominated in the narratives analyzed. These tenses situate the narrative and guide the narrated worlds toward distant events._^17^_

Moreover, hearing-impaired children's difficulties relating to form, content and use give rise to inadequacy of narrative competence regarding proposition use, narrative scores, narrative cohesion, cohesion measurements and overall narrative scores, with associations with the children's ages and the type of school attended (public or private)._^4^_

Finally, our data suggest that these hearing-impaired children undergoing speech and language therapy showed self-organization with regard to time, space and causal relationships, because they were able to put events into a sequence according to temporal succession. The acquisition of oral language does not just happen suddenly: it is a time-dependent process. To be linguistically competent, some form of linguistic input should appear as early as possible in their lives._^5^_

Although studies have been conducted with the aim of understanding the difficulties and the nature of language acquisition and development among deaf children, only a few have described the process of word acquisition in this population. For this reason, findings relating to how this process occurs in deaf children would facilitate early intervention by healthcare personnel aimed towards optimizing language skills during therapeutic interventions, so that these children would be able to enter school with an adequate level of spoken language later on.

### CONCLUSION

Among oralized hearing-impaired children who underwent speech-language therapy, their use of verbs and nouns was similar to that of their hearing counterparts. The present data are important for therapeutic planning and also for appropriate inclusion of hearing-impaired children in the educational system and, afterwards, in the job market.
